# Evaluation of the Zero Shear Viscosity, the D-Content and Processing Conditions as Foam Relevant Parameters for Autoclave Foaming of Standard Polylactide (PLA)

**DOI:** 10.3390/ma13061371

**Published:** 2020-03-18

**Authors:** Tobias Standau, Huan Long, Svenja Murillo Castellón, Christian Brütting, Christian Bonten, Volker Altstädt

**Affiliations:** 1 Department of Polymer Engineering, University Bayreuth, Universitätsstraße 30, 95447 Bayreuth, Germany; tobias.standau@uni-bayreuth.de (T.S.); christian.bruetting@uni-bayreuth.de (C.B.); 2 Bavarian Polymer Institute and Bayreuth Institute of Macromolecular Research, University of Bayreuth, Universitätsstraße 30, 95447 Bayreuth, Germany; huan.long@uni-bayreuth.de; 3 Institut für Kunststofftechnik, University of Stuttgart, Pfaffenwaldring 32, 70569 Stuttgart, Germany; svenja.murillo.castellon@ikt.uni-stuttgart.de (S.M.C.); christian.bonten@ikt.uni-stuttgart.de (C.B.)

**Keywords:** polylactide, biofoams, zero shear viscosity, melt strength, batch foaming, pressure drop rate, low density, small cell size

## Abstract

In this comprehensive study, the influence of (i) material specific properties (e.g., molecular weight, zero shear viscosity, D-content) and (ii) process parameters (e.g., saturation temperature, -time, -pressure, and pressure drop rate) on the expansion behavior during the autoclave foaming process were investigated on linear Polylactide (PLA) grades, to identify and evaluate the foam relevant parameters. Its poor rheological behavior is often stated as a drawback of PLA, that limits its foamability. Therefore, nine PLA grades with different melt strength and zero shear viscosity were systematically chosen to identify whether these are the main factors governing the foam expansion and whether there is a critical value for these rheological parameters to be exceeded, to achieve low density foams with fine cells. With pressure drop induced batch foaming experiments, it could be shown that all of the investigated PLA grades could be foamed without the often used chemical modifications, although with different degrees of expansion. Interestingly, PLAs foaming behavior is rather complex and can be influenced by many other factors due to its special nature. A low molecular weight combined with a high ability to crystallize only lead to intermediate density reduction. In contrast, a higher molecular weight (i.e., increased zero shear viscosity) leads to significant increased expandability independent from the D-content. However, the D-content plays a crucial role in terms of foaming temperature and crystallization. Furthermore, the applied process parameters govern foam expansion, cell size and crystallization.

## 1. Introduction

Polylactide (PLA) has drawn enormous attention in the past two decades as one of the most promising alternatives for replacing foamed products made from conventional plastics, such as polystyrene (PS), because of its similar mechanical properties [1] and because of the lower carbon foot print [2]. Though, it is well known that it is very challenging to foam PLA due to its poor melt strength and complex crystallization behavior. Different ways to improve the foamability of PLA were described in the literature and concluded in a work of Nofar et al. [3], as using chain extenders to increase the molecular weight and/or induce branched chains, varying the ratio of the so called L/D enantiomers (i.e., the composition of PLA from chiral L-lactide and D-lactide), using additives and controlling the crystallization behavior.

In the current literature, mainly the use of a wide variety of chemical modifications during processing and foaming is mainly described. Mostly, these modifiers are added to increase the molecular weight and prevent degradation. This can lead to chain extension, chain branching and/or cross-linking. Typical modifiers that are used in combination with PLA can be distinguished by the occurring reaction into two groups: (i) functional group reactions and (ii) radical reactions and have been summarized in our previous work [4]. Additionally, the rheological as well as thermal behavior is influenced by the use of these modifiers [5]. The most common used modifier is the commercial additive Joncryl^®^ from BASF SE, which is a multi-functional epoxy based chain extender that reacts with the functional groups of PLA (i.e., carboxyl- and hydroxyl groups) during melt processing. Peroxides, such as dicumyl peroxides, lead to free radical reaction. In one of our previous studies, we could show that the melt strength could be significantly increased by the use of these modifiers, leading to foams with lower density, finer cell sizes and improved compression behavior [6,7].

Even though it is often stated in literature that the melt strength plays a vital role in the foaming of PLA, only a few publications actually quantify it at all [7,8,9,10]. An increased melt strength is beneficial for foaming, as it prevents coalescence and rupture during the cell growth step because the cell walls can withstand higher forces during stretching while expanding and can consequently lead to an improved expansion behavior (i.e., lower density and finer cells) [11].

The zero shear viscosity (ZSV) can be obtained from simple shear experiments with less experimental effort, when compared to the determination of the melt strength. The ZSV is dependent on a lot of different factors, such as molecular weight, modifications [12], chain architecture [13,14], and the amount of plasticizing agents, additives [15], or other blend partners [16]. Therefore, the zero shear viscosity can be quite easily varied. For example, Najafi et al. [17] investigated the rheological properties and foaming behavior of modified polylactide. Within this study, a semi-crystalline linear polylactide was modified with an epoxy-based chain extender. Here, the zero shear viscosity was increased and strain hardening was induced. It was shown that a higher shear viscosity in combination with the strain hardening lead to a finer cell morphology and lower foam densities. A recent work by Nofar et al. [18] dealt with rheological, thermal, and foaming behavior of unmodified and modified PLA. In both of the studies, the modification of PLA led to an increase in molecular weight and introduced a strain hardening effect at the same time. For this reason, it was not possible to separate the effect of the molecular weight from the strain hardening on the foaming behavior. Up to now, no systematic work on the influence of the zero shear viscosity on the expansion behavior of PLA was carried out. Yet, it is not clear from the literature whether a certain zero shear viscosity needs to be exceeded to obtain low density foams.

Besides the rheological behavior, the crystallinity is a key factor for processing PLA. The monomer lactic acid is chiral, thus there are two lactide isomers: L-Lactide and D-lactide. Here, the first is commonly the major component of commercial PLA grades usually with a content that is above 90%. The ratio between L-Lactide and D-lactide (L/D ratio) governs the chain architecture, hence the crystallization behavior of PLA. It should be mentioned that PLA with D-content above 10 to 12% are hindered to crystallize [19]. It is already known that PLA (with a D-content below 10%) usually crystallizes during foaming [20]. If PLA crystallize, multiple modifications are known, which occur under varied conditions; the detailed description was summarized in our previous work [4]. Besides the chain architecture, the crystallization behavior of PLA is also strongly dependent on the molecular weight itself, as the elevated molecular weight would restrict the chain mobility. During the pressure-induced batch foaming process, the crystallization behavior of PLA can be assumed as being quite complex and the following processing parameters strongly impact the final foam properties:

(1) In general, temperature most directly affects the crystallization behavior by affecting chain mobility.
(2) The pressure dominates the solubility of the blowing agent and, thereby, the amount of it that is solved in the polymer matrix. Hence, the plasticization effect as well as the gas-induced crystallization is directly influenced by the applied pressure.
(3) During an isothermal saturation phase (time), the crystallization kinetics of PLA is promoted by elevated temperature and the plasticization effect from dissolved blowing agent and is generally governed by the duration.
(4) As a consequence of a sudden pressure drop, which actually induces a thermo-dynamic instability and the prompt oversaturation of the sample the foaming takes place. Hence, the pressure drop rate guides the nucleation rate and the extent of the strain-induced crystallization.

This study focuses on the foamability of the neat (i.e., unmodified) PLA, carving out which requirements for high expansion and fine morphology in an autoclave foaming process are necessary. By the comparison of a wide variety of commercial grades, different aspects, such as D-content, molecular weight, and rheological behavior, can be evaluated regarding their influence on the foaming behavior. Here, besides the often referred melt strength, the zero shear viscosity is also about to be considered as foam relevant property. Additionally, the processing parameters themselves were studied and correlated with the material-specific parameters. Yet, novel cross-relationships could be revealed, as material- and process-specific factors were systematically varied over a wide range.

## 2. Materials and Methods

### 2.1. Materials

In this study, different PLA grades from NatureWorks Ltd. (Minnetonka, MN, USA) were used. Table 1 lists the selected grades and their supposed usage, as well as the melt flow rate from the data sheet. Additionally, the D-content, the molecular weight, and the zero shear viscosity are given.

### 2.2. Methods

#### 2.2.1. Size Exclusion Chromatography (SEC)

The SEC measurements were performed on a 1260 SECcurity by PSS (Mainz, Germany) that was equipped with a refractive index detector. A 50 mm precolumn and three 300 mm PSS SDV 10 µm columns with 103, 105, and 106 Å were used as stationary phase. Chloroform was used as a mobile phase. The column temperature was set to 30 °C and the flow rate was 1.0 mL/min. The sample concentration was 3.0 g/L. Chromatograms were interpreted while using conventional calibration against polystyrene polymer standards. Therefore, please mind that the noted molecular weights are not the absolute values and can only be compared within the test series.

#### 2.2.2. Differential Scanning Calorimetry

The thermal properties of the samples were analyzed by differential scanning calorimetry (DSC) on a DSC 204 Phoenix from Netzsch (Selb, Germany). Samples of about 10 mg were weighed and then sealed in DSC crucibles and placed in the DSC cell. The measurements were performed under nitrogen atmosphere. First, they were heated from 20 to 200 °C at 10 K/min. Subsequently, they were cooled down from 200 to 20 °C with 10 K/min. and then heated up again from 20 to 200 °C at 10 K/min. Additional experiments with elevated heating rates of 20 K/min. were also carried out. It is well known that the crystallization of PLA is very slow. Hence, measurements were also performed at a lower cooling rate of 2 K/min to further investigate the crystallization process. The crystallinity of the materials was calculated by the use of heat of fusion of 93.1 J/g for 100% crystalline PLA homopolymers [22].

#### 2.2.3. Rheology

(1) Complex Viscosity: The rheological characterization in shear flow was performed with a plate-plate rheometer Discovery HR-2 hybrid from TA Instruments Waters LLC (New Castle, DE, USA) at 180 °C with a diameter of 25 mm and a gap of 1 mm under nitrogen atmosphere. Dynamic mechanical experiments were carried out in a frequency range from 500 to 0.01 rad/s. The deformation amplitude was set to 5% for all of the measurements. The zero shear viscosity was determined in the frequency region of the Newtonian plateau at a frequency of 0.1 rad/s.
(2) Melt Strength: The Rheotens measurements were carried out to prove the ability of the polymer melt to withstand uniaxial strain. The melt strength was measured while using a Göttfert Rheotens device (Buchen, Germany). The melt strength test samples were prepared using a single screw extruder (L/D ratio of 26) from Göttfert (Buchen, Germany), equipped with a round die of 6 mm in diameter. For each test, a molten polymer strand was drawn down from the die by the two counter-rotating measurement wheels that were mounted on a sensitive force transducer connected to the Rheotens Göttfert 71.97 unit. The tensile force on the polymer melt strand was measured as a function of time or velocity of the measurement wheels. The melt strength is represented by the force at which the strand breaks or in the plateau phase of the rheotens curve. The measurements were carried out at 180 °C and a constant acceleration of 2.4 mm/s^2^.

#### 2.2.4. Autoclave Foaming

**Figure 1:**
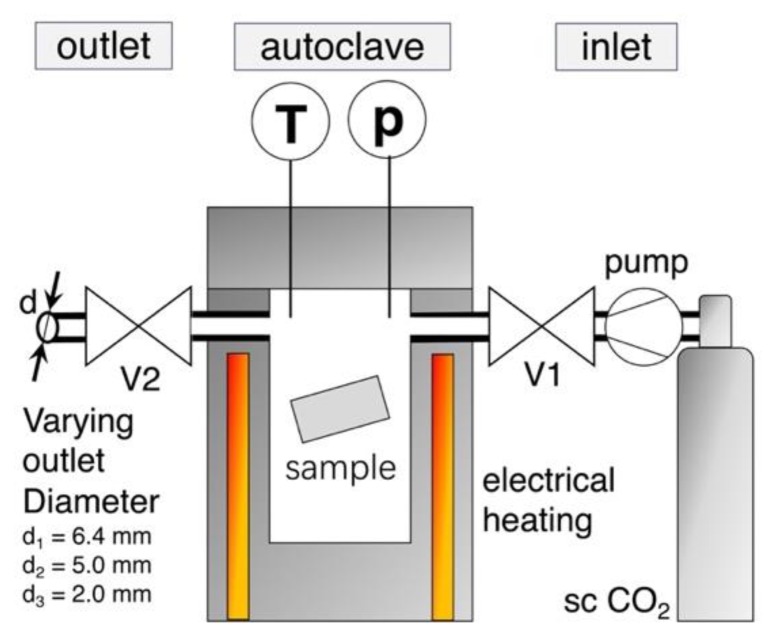
Sketch of foaming set up with the custom-made autoclave.

Foaming was carried out with the pressure drop method on a custom-made autoclave, which Figure 1 schematically illustrates. The melt pressed samples (10 × 20 × 1 mm^3^) were put in the electrical heated pressure vessel, which was then purged with CO_2_.

By the use of a twin set-up of Teledyne Isco 260D syringe pumps (Thousand Oaks, CA, USA), supercritical CO_2_ was applied and the samples were impregnated with a constant saturation pressure of 180, 150, or 120 bar, respectively. The pump was set to constant pressure delivery mode, which ensured a constant pressure during the whole saturation period (even at small leakages). Temperature is measured in the vessel near the sample and it was varied over a wide range for each grade, to find optimum foaming condition (i.e., minimum in foam density). The saturation time was set as 30 min. (but also was varied for selected grades between 5 min. and 8 h). After saturation, the electrical controlled pneumatic valve V2 was promptly opened to start the pressure drop, which initiates foaming. Reproducibility is given for the out carried experiments, as the threefold repetition of a foaming trial usually resulted in standard deviations that were below 5%.

**Figure 2:**
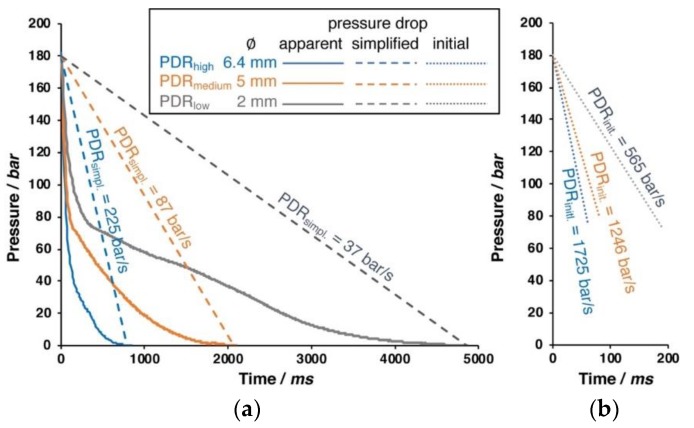
Different pressure drops that can be realized with different pipe diameters. (**a**) Apparent pressure drop (solid line) and simplified linear pressure drop (dashed line). (**b**) Close up of the initial pressure drop (dotted line) during the first milliseconds.

By changing the inner diameter of the outlet pipe (6.4, 5.0, or 2.0 mm), the pressure drop rate can be varied, as shown in Figure 2.

Only the initial phase of the pressure drop is nearly linear, as can be seen in the right close up in Figure 2. The apparent pressure follows a logarithmic function. The pressure drop rate (PDR) that results from different pipe diameters is also noted within the diagram. For the simplified pressure drop rate, a linear drop is assumed over the entire time. However, this is only a very rough description; and, from visualization experiments in foam extrusion process carried out by the group of C.B Park, it is known that the cell formation (i.e., cell nucleation) takes place in the first milliseconds of the pressure drop [23]. Therefore, the initial pressure drop rate is calculated from the linear slope during the depressurization to 90 bar happening in 50 ms (6.4 mm), 90 ms (5.0 mm), and 190 ms (2.0 mm), respectively. If not given otherwise, the experiments were carried out with 180 bar saturation pressure and the largest outlet of 6 mm diameter (i.e., with the highest pressure drop rate). The pressure drop rate is suggested to have impact on the foaming behavior by affecting the nucleation and cell growth [24,25]. At high pressure drop rates, the nucleation is faster, because the thermodynamic instability, which is responsible for the cell formation, happens earlier. Besides, the cell growth is also faster, while the diffusive path of dissolved gas to reach a cell is shorter with the increasing amount of nuclei. In the literature, pressure drop rates rarely can be found. Liu et al. reported 1.3 MPa/s (converts to 13 bar/s) as the pressure drop rate during autoclave foaming trials [26]. In their work, Xu et al. [27] mention that the pressure drop is not constant over the time, but quantify it by an average, as the ratio of pressure difference and depressurization time (i.e., simplified, as dashed line in Figure 2). By varying the PDR from 1.4 to 25 MPa/s (14 up to 250 bar/s), they showed that a higher PDR leads to increased cell density and a higher volume expansion ratio.

#### 2.2.5. Foam Properties

(1) Scanning Electron Microscopy (SEM): Cryogenic fractured foam samples were investigated by SEM JEOL JSM-6510 (Akishima, Japan). The cell sizes were determined by the use of image analysis software (ImageJ, v1.48, University of Wisconsin, Madison, WI, USA).
(2) Density: The density was determined according to the Archimedes principle with a balance from Mettler Toledo AG245 (Columbus, OH, USA).

## 3. Results and Discussion

### 3.1. Basic Properties of the Neat Materials

First, the basic properties of the neat materials were determined in order to be able to correlate the molecular constitution (molecular weight and D-content) with the rheological and thermal properties, as well as the later foaming behavior.

The Rheotens test was performed to evaluate the influence of the melt strength on the foaming behavior. It was expected that the melt strength increases with higher molecular weight, due to an increase of polymer chain entanglements. Entanglements can lead to forces that are similar to secondary or hydrogen bonding forces. Therefore, the resistance to an applied pulling load (here draw down force) is higher with a higher molecular weight. In regards of foaming, it is expected that a certain melt strength is necessary to build a stable foam structure [28].

**Figure 3:**
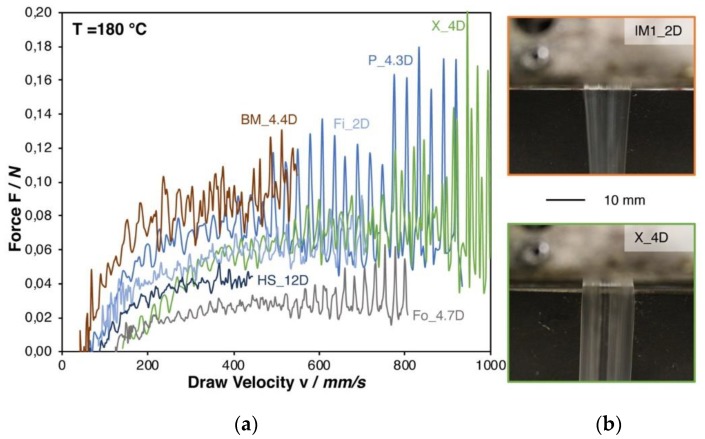
(**a**) Rheotens curves of all (measurable) PLA grades at 180 °C. (**b**) Die swelling behavior of a low and a high molecular PLA.

Figure 3a shows the Rheotens curves. Even though both grades have a molecular weight in a similar range (see Table 1), the melt strength of IM1_2D was not measurable, while it could be determined for PLA Fo_4.7D, which shows a melt strength of 0.03 N. It has to be pointed out that the injection molding grades (IM1_2D, IM2_1.4D, and IM3_2D) could not be successfully measured due to the occurring strand rupture already at very low strain. This is caused by the comparable low viscosity at the measuring temperature. As a consequence, a pronounced melt strand thinning is caused by gravity forces. This effect can be seen in Figure 3b, while comparing IM1_2D and the higher molecular weight grade X_4D.

Yet, the melt strength can be a meaningful value to be correlated with the foamability, it can only be determined for selected grades. The shear behavior was also measured to generate more reliable information on all grades.

**Figure 4:**
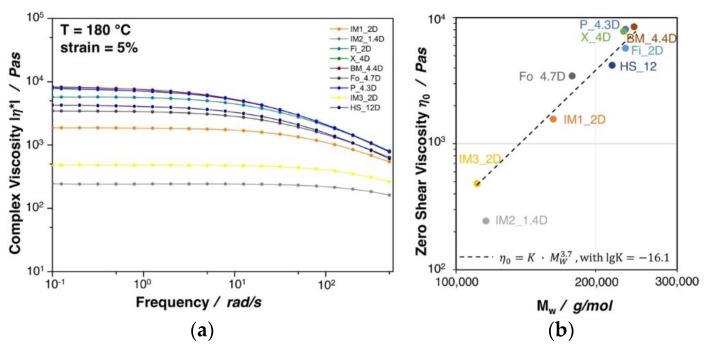
(**a**) Complex viscosity of neat PLA grades at 5% strain and 180 °C. (**b**) Zero shear viscosity plotted against the molecular weight.

Figure 4a shows the complex viscosity as a function of the frequency for the investigated PLA grades. All of the materials show a typical shear thinning behavior, which is more pronounced with increasing complex viscosity. A direct correlation between the zero shear viscosity (complex viscosity at a frequency ≈ 0.1 rad/s) and the molecular weight was found, as can also be seen in Figure 4b. Besides a slight deviation of IM2_1.4D, for all grades the zero shear viscosity is proportional to the 3.7th power of molecular mass, thus confirming the linear character of the polymer chains [29,30]. A higher molecular weight and/or the existence of long chain branches are beneficial to achieve a high expansion during foaming, as it was shown for PP extrusion foams in the work of Stange et al. [31]. It can be seen that the two injection molding grades IM3_2D and IM2_1.4D possess a rather low complex viscosity, which is clearly due to their very low molecular weight. Similar to the melt strength, the complex viscosity, in general, increases with increasing molecular weight. Though, a direct correlation between the molecular weight and the melt strength was not possible due to the influence of various factors (external: e.g., temperature shifts over the down drawn melt strand and internal: e.g., different crystallization rates).

**Figure 5:**
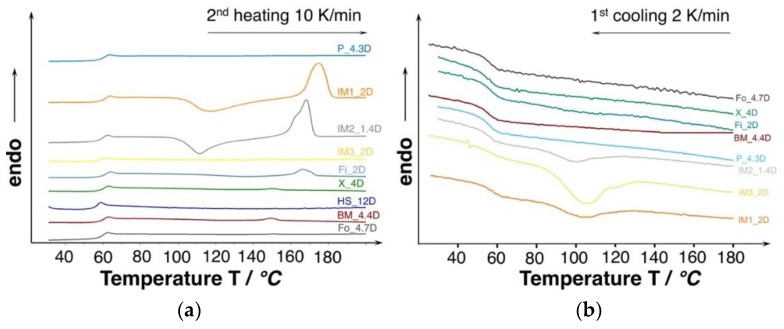
(**a**) Differential scanning calorimetry (DSC) curves of unprocessed PLA (10 K/min., 2^nd^ heating). (**b**) Cooling curves of the unprocessed PLA with very low cooling rate to favor crystallization (2 K/min.).

The molecular weight and D-content [32] strongly affects the thermal properties (i.e., crystallization behavior). The DSC thermograms of the investigated PLA are shown in Figure 5. Judging from the second heating curves, all of the grades only revealing a neglectable amount of crystallinity (0.3–3%) and they can be considered as amorphous. However, the injection molding grades show the tendency of cold crystallization.

In general, PLA show a very slow crystallization rate and most of the grades are not able to crystallize while the cooling phase during the DSC experiment, even at very slow cooling rates of 2 K/min. (see Figure 5b). However, the injection molding grades were able to crystallize partly already during the cooling. The ability to crystallize already during the cooling can be affiliated to the lower molecular weight, resulting in higher chain mobility. Consequently, higher crystallinities could be expected under certain conditions, allowing for PLA chains to come close enough to build ordered structures (i.e., crystals), such as sufficient time and/or a high strain. Additionally, it would be likely that the manufacturer already incorporated nucleating agent.

The different thermal behavior will also lead to varied crystallization during the foaming. Here, a fast crystallization rate can be beneficial, as formed crystals could increase the melt strength and they can act as nucleating points for cell growth resulting in a stable and fine celled foam structure. However, on the other side, a too high crystallization rate and degree of crystallinity can hinder the cell expansion.

### 3.2. Expansion of the Neat Grades

**Figure 6:**
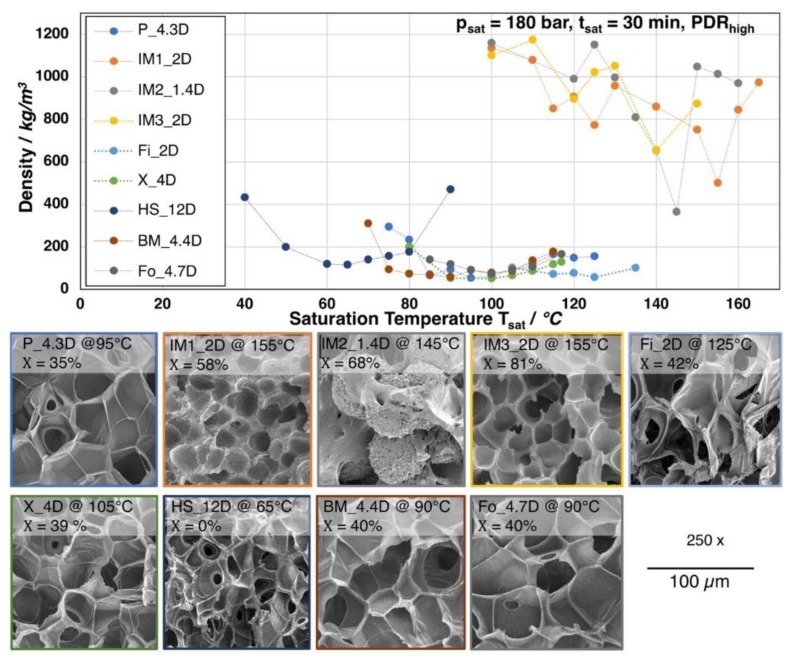
Top: Foam densities of the neat PLA grades foamed after a 30 min. saturation with CO_2_ at 180 bar at varying temperatures. Bottom: SEM images of the foam with lowest density for each grade with crystallinity values (1^st^ heating).

**Figure 7:**
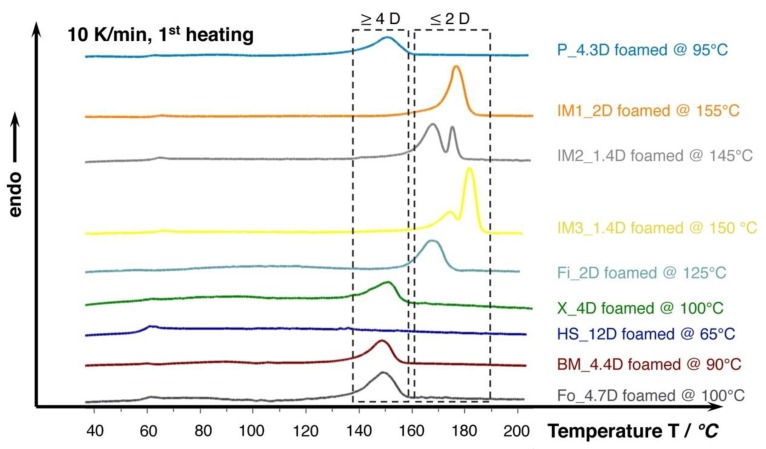
DSC curves (10 K/min., 1^st^ heating) of the lowest density foams achieved in batch foam experiments with 30 min. saturation at 180 bar. Note the increased melting temperatures marked in the dashed boxes of PLA foams with lower D-content of 2 and 4, respectively.

All of the grades were foamed with a saturation time of 30 min. at 180 bar CO_2_ and varying temperatures. In Figure 6, the obtained foam densities, depending on the saturation temperature, are shown. Additionally, a SEM image of the morphology for each grade with the lowest achieved density is given. Most of the grades show an optimum (i.e., minimum of the foam density) at a certain temperature. The lowest densities range between 150 and 50 kg/m^3^. However, the grades IM1_2D, IM2_1.4D, and IM3_2D show much more erratic behavior and increased standard deviation above 5%. Here, no clear trend is visible and foam densities are much higher (above 400 kg/m^3^). The mentioned grades are injection molding grades with low molecular weight, low zero shear viscosity, and a strong ability to crystallize. The melt strength is assumed to be very low, as these grades could not be analyzed by Rheotens experiments. Furthermore, all these types possess a low number average molecular weight M_n_, which indicates short polymer chains, resulting in higher crystallization rates due to a higher chain mobility. Hence, the degree of crystallization of 58% up to 81% (see Figure 7) is found to be much higher after the depressurization, as compared to the other grades. Consequently, the cell growth is hindered during expansion. Two effects are likely: (i) the sample starts to crystallize in the presence of the CO_2_ during the saturation, which could lead to a reduced gas uptake by the developed crystalline phases [33,34] and/or (ii) shorter chains can be oriented in a much higher extent during the stretching, while the expansion (so called strain induced crystallization) [20]. Additionally, as these grades are commercialized, they are most probably additivated with nucleating agents by the manufacturer. Also other studies have only revealed a moderate density reduction for batch foamed PLA injection molding grades [35,36].

Apart from the injection molding grades, for P_4.3D, Fi_2D, X_4D, BM_4.4D, and Fo_4.7D foams with densities below 100 kg/m^3^ were achieved at significant lower foaming temperatures, as is clearly visible from Figure 6. These five grades mainly possess a higher molecular weight and, consequently, higher melt strength, as well as higher zero shear viscosity, which allows for a more pronounced expansion. Thus, the cell growth could also be ensured. However, IM1_2D and Fo_4.7D owe molecular weights in a similar range (see Figure 4b) but they show much different foaming behavior, which allows for the conclusion that the expansion is affected by other parameters beside the molecular weight, such as the crystallization rate and amount of crystals, which appears to be much higher for the injection molding grade IM1_2D. The lowest achieved densities are 40 kg/m^3^ (BM_4.4D), 52 kg/m^3^ (P_4.3D), 87 kg/m^3^ (X_4D), and 71 kg/m^3^ (Fo_4.7D). Even though the first three mentioned grades possess a higher molecular weight and zero shear viscosity than Fo_4.7D, they all show similar expansion. Consequently, it can be stated that the molecular weight and the therefrom resulting rheological behavior seems to be sufficient enough to allow for the formation of low density foams, as the expansion is not hindered by a too strong crystallization, as it would be the case for IM1_2D. After foaming, the grades P_4.3D, Fi_2D, X_4D, BM_4.4D, and Fo_4.7D also developed significant elevated crystallinities. The degree of crystallinity ranges from 35% up to 42%, which is still lower than for the injection molding grades (> 58%). The chain mobility is expected to be significantly lower than that for the injection molded grades hindering the development of higher orientation during crystal formation, as these grades have longer chains. It should be mentioned that the crystallization is also assumed to happen during the saturation with the aid of the elevated temperature and chain mobility. Thus, it could be assumed that, underneath a critical extent, the so-formed crystals would increase the melt strength and serve as nucleation points and, thereby, be beneficial during the expansion.

Furthermore, the DSC curves that are shown in Figure 7 reveal that the melting temperature of the crystallized foams seems to strongly depend on the D-content. The grades with D-content of approximately 2% or lower (IM1_2D, IM2_1.4D, IM3_2D, and Fi_2D) show elevated melting temperatures of around 170 °C and higher while the grades with D-content around 4% (P_4.3D, X_4D, BM4.4D, and Fo_4.7D) clearly exhibit a lower melting point of around 150 °C. At higher D- contents, even lower melting points could be expected, as it was shown in the work of Bigg [37] for the case of unfoamed PLA. The formation of double melting peaks was also observed for the grades IM1_2D and IM2_1.4D. This effect is known for PLA and it could also happen to other grades, as it is mainly sensitively influenced by the saturation conditions (temperature, time and pressure), which was well described by Nofar et al. [38].

HS_12D is another outstanding grade, which foams at much lower temperatures. In fact, the minimum of foam density (115 kg/m^3^) is reached at 65 °C. This can be traced back to the much higher D-content of approximately 12. Hence, no crystallization is expected to happen under this conditions [19,22,39] and the foaming temperature depends mainly on the T_g_. The T_g_ of HS_12D could be expected to shift to a lower range [40], as well as the melt strength, due to the plasticization effect of the blowing agent. The molecular weight of HS_12D is between Fo_4.7D and X_4D, which both foamed to lower densities. This allows for the conclusions that the absence of crystallization due to the high D-content results in less favorable rheological behavior for foaming in the autoclave.

Most of the foams possess very thin cell walls. Here, it is evident that some of these cell walls are opened due to cell coalescence and cell rupture. A phenomenon that has been often reported for PLA foams [39,41,42] and can be traced back to the low melt strength.

### 3.3. Foaming Conditions vs. Expansion

**Figure 8:**
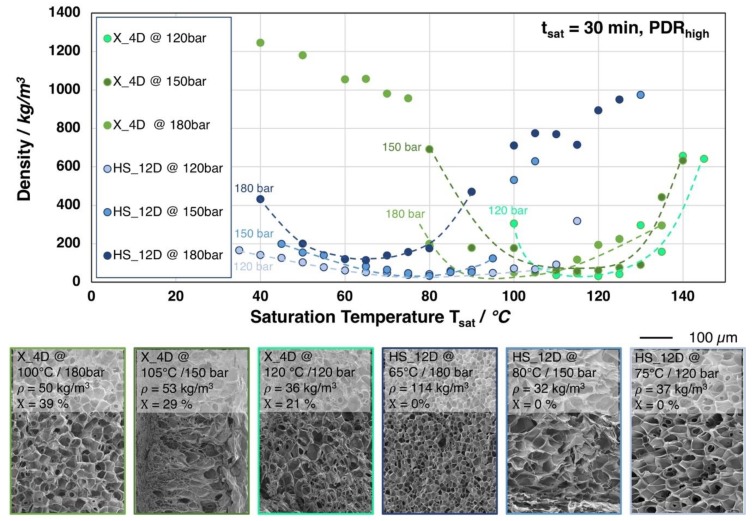
Top: Foam densities of the grades X_4D and HS_12D (with similar molecular weight but varying D-contents) foamed after 30 min. saturation with CO_2_ at 120, 150, and 180 bar and varying temperatures. Bottom: SEM images of the foam with lowest density for both grades with crystallinity values (1^st^ heating).

In Figure 8, the influence of the saturation pressure on the achievable foam density is shown for X_4D and HS_12D (similar molecular weight but different D-content). Surprisingly, at lower saturation pressures, foams with lower densities but larger cell sizes were obtained.

For both grades, it was observed that the saturation temperatures required for achieving the lowest possible foam densities are generally shifted to higher values with lower saturation pressure. This is a result of a less pronounced plasticization effect with a lower amount of dissolved CO_2_ at lower saturation pressure. Moreover, HS_12D showed more pronounced foam density drop with lower saturation pressure and no crystallinity was observed after foaming. This could only be explained, as (i) a high expansion was ensured by a higher melt strength and (ii) that the pressure drop (rate) itself reduces with decreasing saturation pressure, thus leading to a lower cell density, as it is also described in literature [24,25]. Hereafter, the cells have the chance to grow larger and, consequently, result in lower foam density. Moreover, the gas dissolving content is considered also to have an effect on the cell nucleation. Guo et al. [43] described that an elevated saturation pressure promotes the nucleation rate; hence, a larger cell size can normally be expected from a lower saturation pressure. This is confirmed by the SEM images in Figure 8.

Similar tendency was observed for X_4D—yet much weaker—which is assumed to be mainly influenced by its thermal properties. Since X_4D possesses much lower D-content than HS_12D and showed crystallinity after foaming, the formation of crystals could be expected during CO_2_ saturation. However, with less gas dissolving under lower pressure and at elevated saturation temperature, the crystallization was less promoted, leading to lower crystallinity values of 29% (at 150 bar) and 21% (at 120 bar), respectively.

**Figure 9:**
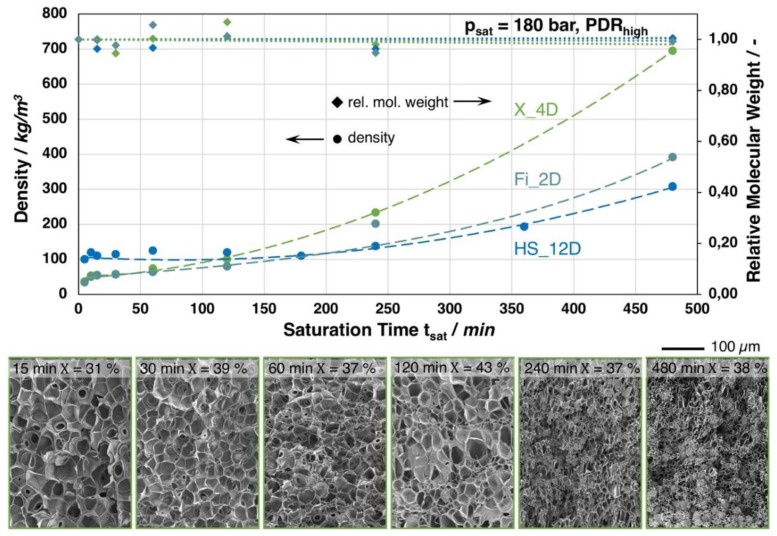
Top: Foam densities of the grades Fi_2D, X_4D, and HS_12D (with similar molecular weight but varying D-contents) foamed with CO_2_ at 180 bar and varied saturation times. Bottom: SEM images of the X_4D foam with different saturation times and crystallinity values (1^st^ heating, 10 K/min.).

In Figure 9, the foam density in dependence of the saturation time is shown for the grades Fi_2D, X_4D, and HS_12D. The foaming experiments were carried out at the optimum foaming temperatures for each grade (based on the trials summarized in Figure 6). Unusual high saturation times of up to eight hours were chosen here to identify the cause for the relenting density reduction with increasing saturation time. One could expect that a longer saturation time would lead to a higher expansion. Hence, for all grades, the lowest densities were achieved between 10 and 30 min. of saturation, respectively. For longer saturation, the achieved foam density increases almost exponential, as it can be seen from the trendlines. For the short saturation, a monomodal cell size distribution can be seen from the SEM images of X_4D. With increasing saturation times, a bimodal cell size distribution with much smaller cells is visible. After 8 h saturation, the morphology is comparable to IM2_1.4D (145 °C, 180 bar, 30 min.; cf. Figure 6), showing scattered spherical structures in between the cells.

The explanation for the decreased expansion with longer saturation time is not as trivial as expected. Of course, a decrease in molecular weight could be assumed with longer saturation to explain the decreasing expansion from the consequently anticipated less favorable rheological behavior during the expansion. However, the samples do not undergo any significant thermal degradation during saturation at different temperatures, as it can be clearly judged by the GPC results. Here, the relative molecular weight, as well as the polydispersity, did not significantly change. Another possible explanation would be that the absorption is ongoing over the time, meaning that the samples are not fully saturated with CO_2_. The consequence would be that the T_g_ (and also the viscosity) would be decreased by a more pronounced plasticizing effect with increased saturation time, meaning that the optimum temperature for foaming would shift to ever lower values. Repeating the experiments at a lower temperature could impose this, which did not lead to lower densities. This allows for the conclusion that the samples can be assumed as being fully saturated, even after short saturation times and that there must be another reason for the less pronounced density reduction with an increasing saturation time. It is striking that HS_12D shows slightly divergent behavior when compared to the other grades (with lower D-content), as the density does not change significantly up to a saturation time of 240 min. When considering this and the crystallinity values (cf. Figure 9) of the foams from Fi_2D and X_4D, we develop the hypothesis that crystallization seems to play a crucial role for the expansion at different isothermal saturation under high pressure CO_2_ loading (even for PLA with high D-content, which is strongly hindered to form crystals). There is strain-induced crystallization during the foaming, as was mentioned earlier. This leads to relatively high crystallinity values with high expansion rates. Henceforth, it would be assumed that, with less expansion, less (strain induced) crystallinity would be seen. Nevertheless, the values for the long saturated but less expanded samples stay rather high. Yet, this can be explained by the CO_2_ induced crystallization, as was described by Xu et al. [44], showing elevated crystallinity for PLA saturated with super critical CO_2_ for more than ten minutes. This allows for the assumption for Fi_2D and X_4D, that the CO_2_-induced and strain induced crystallization are overlapping effects and with ongoing saturation the CO_2_-induced crystallization will prevail. Hence, the crystals that formed during the saturation can hinder the expansion during depressurization, as the rheological behavior can be expected as being less favorable for expansion.

**Figure 10:**
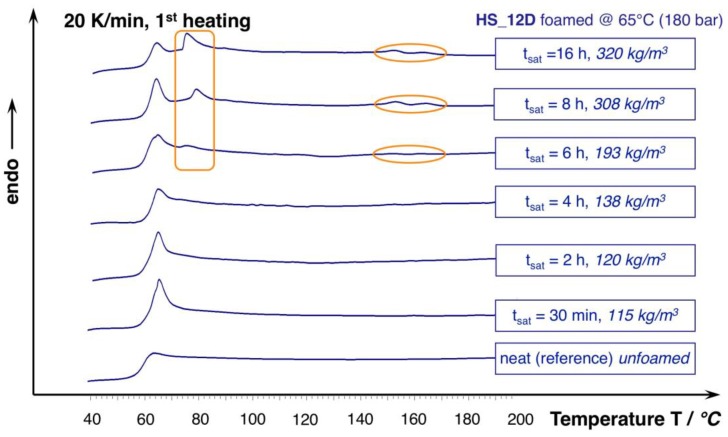
DSC curves of HS_12D foams and unfoamed reference (1^st^ heating, 20 K/min.).

For HS_12D, this effect does not seem to play a role, which is plausible, as it is considered as total amorphous. However, this only seems to be valid for the saturation approximately up to 240 min. The DSC curves from the foams that were saturated longer reveal an additional phase transition that is close to the T_g_ and the formation of slight melting peaks at 153 and 166 °C (see Figure 10), which could be enabled from the harsh conditions of the applied high pressure and the long saturation phase at elevated temperature. After 16 h, this effect levels out, as judged from the associated density change. Hence, it is not unlikely that the autoclave treatment of usually amorphous polymers results in crystallization, as it was also shown before for polycarbonate [45]. To further describe the responsible mechanisms, more advanced techniques (e.g., high pressure DSC, modulated DSC, rheology of gas loaded melt, wide-angle X-ray scattering WAXS) would be helpful, but they could not be applied within this study. Still, it can be concluded that a long saturation phase under isothermal conditions leads to crystallization that is favored by the presence of CO_2_, which is not beneficial for the expansion; and, even PLA with high D-content, which can normally be considered as totally amorphous is affected, as it shows additional phase transitions.

**Figure 11:**
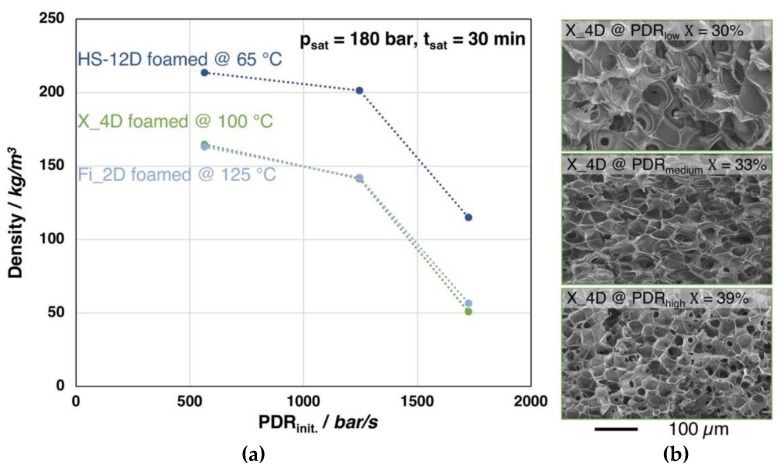
(**a**) Foam densities of the grades Fi_2D, X_4D and HS_12D (with similar molecular weight but varying D-contents) foamed after 30 min. saturation with CO_2_ at 180 bar and varied pressure drop rates. (**b**) SEM images of the X_4D foams with different pressure drop rates and crystallinity values (1^st^ heating).

The foaming behavior (i.e., density) of Fi_2D, X_4D, and HS_12D, depending on the pressure drop rate, is shown in Figure 11. The foaming experiments were carried out under the optimal conditions of each grade (i.e., saturation time, -temperature, and -pressure based on the findings of Figure 6), yet different gas outlet pipe diameters were used to vary the PDR.

Clearly, density reduction is more pronounced with an increasing pressure drop rate for all the three investigated grades. It should be noted that a similar tendency could be found in all three PLA samples with different D-content, which indicates that PDR plays a similar role in the foaming of PLA, regardless of D-content. However, for the low D-content grades (i.e., Fi_2D and X_4D), the crystallinity is also increased, most likely as the strain induced crystallization becomes more obtruded with the higher expansion. Furthermore, as it can be seen from the SEM images of the foam structures of X_4D, clearly the cell size becomes decreased with increased pressure drop rate, as the nucleation is promoted. As a conclusion, it can be said that the result shows a clear trend, where a higher PDR generally leads to a lower foam density and smaller cell size, as cell nucleation is promoted [43,46].

## 4. Conclusions

A wide variety of commercial PLA grades was investigated in this work. The grades can firstly be distinguished by their molecular weight, which leads secondly to different rheological behavior (i.e., melt strength and zero shear viscosity) and varied thermal properties (i.e., crystallization kinetics). Additionally, different D-contents were considered, which also determine the thermal properties (i.e., crystallinity). Besides these material-specific properties, different process parameters were changed during the pressure induced batch foaming (i.e., saturation temperature, -pressure, -time, and pressure drop rate). Consequently, a wide variety of factors that could influence the expansion behavior of PLA were conclusively studied.

Low density foams (volume expansion ratio VER ≥ 10) could be achieved for most of the PLA grades. Exceptions were the injection molding grades, whose low molecular weights lead to lower zero shear viscosities, vanishing low melt strengths, and high crystallization kinetics, which, in total, limit the expansion. It also could be shown that the rheological properties (melt strength and zero shear viscosity) are not the only factors governing the expandability as the crystallization behavior, which is known to be very complex for PLA can tremendously hinder the expansion, as it is the case for the injection molding grades. The D-content governs the foaming temperature and the melt temperatures of the crystallized foams. PLA with lower D-content needs to be foamed at higher temperatures, as it was also observed that a D-content of approx. 4% leads to melting temperature of around 150 °C, while at 2% and lower it is around 170 °C. A D-content of 12% commonly leads to no crystallization and consequently to much lower foaming temperatures that were way below 100 °C. The saturation pressure strongly governs the foaming temperature on which the lowest density can be achieved, as well as the nucleation, and thereby the fineness of the cells. Surprisingly, a shorter saturation time leads to lower densities, because the CO_2_-induced crystallization intensifies with ongoing saturation, allowing for less expansion, as we hypothesized. Even for PLA with a high D-content of 12%, which can usually be considered as totally amorphous, a divergent foaming behavior (t_sat_ > 4 h) was shown, which is presumably due to an additional phase transition, induced by the long saturation. Furthermore, a high pressure drop rate is favorable for achieving low density foams with fine morphology, as the nucleation rate is increased independently from the D-content.

## Figures and Tables

**Table 1:** Overview of Polylactide (PLA) grades used in this study.

Grade & Supposed Usage * (Data Sheet)	Notation	MFR * (g/10 min.) (@ 210 °C, 2.16kg)	D-Content * (%)	GPC Data ** M_w_ (10^3^ g/mol); M_n_ (10^3^ g/mol); PDI (-)	ZSV ** T = 180 °C, *γ* = 5%, 0.1 rad/s (Pas)
2003D Packaging	P_4.3D	6	4.3 [4]	232; 134; 1.73	8089
3100HP Injection Molding	IM1_2D	24	<2 [21]	162: 107; 1.51	1869
3251D Injection Molding	IM2_1.4D	80	1.4 [4]	116; 78; 1.49	243
3260HP Injection Molding	IM3_2D	65	<2 [21]	111; 75, 5; 1.47	483
4032D Film	Fi_2D	−	1.4–2.0 [4]	232; 149; 1.55	5716
4044D Extrusion	X_4D	−	~4 [21]	230; 117; 1.97	7794
4060D Hot Sealing	HS_12D	−	12–12.3 [4]	217; 118; 1.84	4200
7001D Injection Stretch Blow Molding	BM_4.4D	6	4.4 ± 0.5 [4]	242; 142; 1.71	8472
8052D Foaming	Fo_4.7D	14	4.7 [4]	178; 109; 1.64	3457

* taken from data sheet or literature. ** measured within this work, please check 2.2 Methods and Figure 4.
